# *POC1B*-associated cone-rod dystrophy with bilateral optic disc swelling: A novel clinical observation

**DOI:** 10.1016/j.ajoc.2025.102456

**Published:** 2025-10-22

**Authors:** Noha Bamardouf, Abdulrahman Alsaidi, Faeeqah Almhmoudi, Nooran Badeeb, Enam Danish

**Affiliations:** a College of Medicine, King Abdul-Aziz University, Jeddah, Saudi Arabia; b Department of Ophthalmology, Ministry of National Guard Health Affairs, Jeddah, Saudi Arabia; c Department of Ophthalmology, King Fahd Armed Forces Hospital, Jeddah, Saudi Arabia

**Keywords:** Inherited retinal degeneration, Cone dystrophy, Cone-rod dystrophy, *POC1B*, Bilateral optic disc swelling

## Abstract

**Purpose:**

To report a series of genetically confirmed cone and cone-rod dystrophy cases associated with POC1 Centriolar Protein B (*POC1B*) mutations, including two cases presenting with bilateral optic disc swelling.

**Observations:**

In the first family, three affected siblings had a homozygous nonsense variant in POC1B (NM_172240.3:c.672C > G; p.Tyr224∗), classified as pathogenic. Two of these cases presented with bilateral optic disc swelling confirmed by fundus examination and optical coherence tomography, accompanied by features consistent with cone dysfunction on full-field ERG. In the second family, three affected siblings carried a homozygous frameshift variant in POC1B (NM_172240.3:c.991del; p.Arg331Glufs∗13), also classified as pathogenic. These patients demonstrated early-onset visual loss, photophobia, and cone dysfunction without optic disc swelling. All identified variants were consistent with autosomal recessive inheritance and validated by Sanger sequencing.

**Conclusions and importance:**

Bilateral optic disc swelling is a rare manifestation in COD/CORD and is rarely reported in association with *POC1B* variants. Our cases expand the phenotypic spectrum of *POC1B*-associated retinopathies and highlight the importance of considering optic disc swelling as a possible, albeit rare, feature in COD/CORD.

## 1 Introduction

Inherited retinal degenerations (IRDs) represent a diverse group of diseases characterized by significant genetic and phenotypic variability. These disorders are commonly associated with progressive photoreceptor dysfunction and gradual vision loss, making them a leading cause of visual impairment globally.^1^ Among IRDs, progressive cone dystrophies (CODs) and cone-rod dystrophies (CORDs) stand out due to their distinct patterns of photoreceptor involvement. CODs primarily involve cone degeneration, which may later progress to rod dysfunction, whereas CORDs typically feature both cone and rod dysfunction early in the disease course. Clinically, these disorders manifest as macular disease or diffuse retinopathy with predominant macular involvement, leading to reduced visual acuity, central visual field defects, and color vision disturbances.^2^

The estimated worldwide prevalence of CODs and CORDs ranges from 1 in 20,000 to 100,000.^3^^,^^4^ Advances in molecular genetics have identified mutations in at least 32 genes implicated in these conditions, with GUCA1A, PRPH2, ABCA4, and RPGR among the most extensively studied.^3^^,^^4^ The *POC1B* gene encodes a centriole-associated protein essential for cilia function in photoreceptor cells. Mutations in *POC1B* impair photoreceptor sensory cilia formation, leading to autosomal-recessive cone and cone-rod dystrophies.^5^

Retinal dystrophies are notably prevalent in Saudi Arabia, largely due to the high rate of consanguinity, which is estimated at approximately 58 %—among the highest in Arab countries.^6^^,^^7^ Specific genetic mutations such as RPGRIP1, linked to cone-rod dystrophy 13, and KCNV2, associated with retinal cone dystrophy 3B, have been identified in this population.^6^

The clinical manifestations of CODs and CORDs vary. CODs are characterized by central vision loss, photophobia, and color vision deficits, often without nyctalopia, distinguishing them from CORDs, which involve earlier rod dysfunction and nyctalopia. CORDs also present earlier (mean age 12 years) and progress more rapidly, frequently leading to legal blindness by age 23, compared to 48 years in CODs.^8^, ^9^, ^10^ Interestingly, while optic disc swelling is an uncommon feature of retinal dystrophies, a few case reports have documented its occurrence in rod-cone dystrophy (RCOD), specifically retinitis pigmentosa (RP).^11^, ^12^, ^13^, ^14^ To our knowledge, no studies have reported the incidence of optic disc swelling in a case of COD or CORD.

In this paper, we present a series of genetically confirmed COD and CORD cases secondary to *POC1B* variant featuring the unusual association with optic disc swelling. We also review the existing literature on this phenomenon to provide further insight into its occurrence, implications, and the phenotypic variability of the *POC1B* mutation.

## 2 Materials and methods

This study adhered to the tenets of the Declaration of Helsinki. Written informed consent was obtained from the patients to perform genetic analysis. This study was approved by the Research Ethics Committee of the Medical Services Department for Armed Forces Hospital, Jeddah, Saudi Arabia (IRB approval number: REC 780/2025–17).

### 2.1 Clinical data

A complete ophthalmic examination was performed for all patients, including best-corrected visual acuity (BCVA), intraocular pressure (IOP) measurement, cycloplegic refraction (CR), color vision (CV) using Ishihara plates, anterior segment, and dilated fundus examinations. Multimodal imaging included Optical Coherence Tomography (OCT) (NIDEK; RS-3000 Advance, Japan) of the macula and optic disc, fundus photography and fundus autofluorescence (FAF) using Optomap Panoramic Daytona device (Daytona, Optos, United Kingdom), depending on the patient's cooperation. Full-field Electroretinography (ffERG) was performed using Retimax (CSO, Scandicci, Florence, Italy) according to The International Society for Clinical Electrophysiology of Vision (ISCEV) standards. Visual Field (VF) testing was done using Octopus 900 Perimetry. Multidisciplinary systemic evaluation of hearing, hepatic, renal, and mental functions was also performed.

### 2.2 Genetic testing

Peripheral blood samples were collected from patients and sent to an appropriate laboratory for genetic analysis. Whole-exome sequencing was performed using the MGIEasy Exome Capture V5 probe set and paired-end next-generation sequencing was performed on the DNBSEQ-G400S platform. In-house clinically validated tools and utilities, including a bioinformatics pipeline, base calling, primary filtering of low-quality reads and probable artifacts, and annotation of variants, were applied. The variant filtration and classification were performed in-house. All phenotypically relevant variants of uncertain significance, likely pathogenic, and pathogenic variants in ClinVar, HGMD, as well as all variants with minor allele frequency (MAF) of <1 % in ExAc, ESP, gnomAD, SNP, and in-house databases, in addition to any variant that lies between exons and intron boundaries±20 were considered. CNV predictions were performed using bioinformatic tools, including the Germline CNV Caller from the CLC Genomics Workbench (version 22). All relevant inheritance patterns, based on family history and clinical information, were used to evaluate the identified variants. Genomic variants were used for hg38 assembly. The reported variants were validated using Sanger sequencing. The sequencing was performed with a high overall depth (136x), and 72 reads confirmed the presence of the variants in question.

## 3 Case presentation

### 3.1 Family A

#### 3.1.1 Case 1A (Proband)

**Fig. 1:**
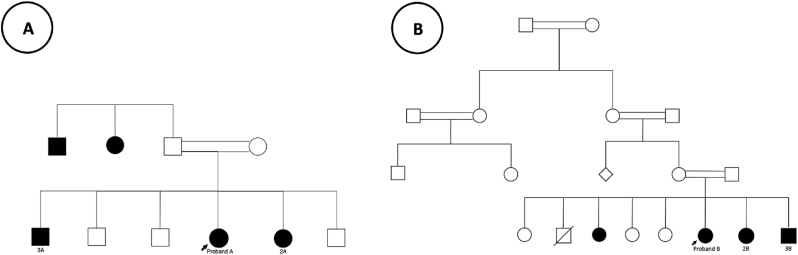
Pedigree chart of family A (Left) and family B (Right) showcasing affected individuals. Some affected individuals were not assessed due to their unavailability for examination, but their inclusion provides broader genetic context.

A 24-year-old female who is known to have polycystic ovarian syndrome (PCOS). She presented with new onset of headache and blurring of vision, which was not associated with nausea, vomiting or tinnitus. Family history was positive for consanguinity, with similar visual complaints reported by her younger sister (2A) and older brother (3A). Additionally, two paternal second-degree relatives were known to have a comparable condition but were not available for assessment (see Fig. 1).

On examination of both eyes, her BCVA measured 20/200 using the Snellen chart. Automated refraction was not significant for refractive errors. Intraocular pressure was 14 and 16 mmHg for the right and left eye, respectively. Color vision testing revealed total color blindness with a score of 0/21. Slit-lamp examination of the anterior segment was unremarkable, while a fundus examination revealed bilateral optic disc edema classified as grade 4 according to the Frisén scale.^15^ Body Mass Index (BMI) was 26. Magnetic Resonance Imaging (MRI) of the brain showed a partially empty sella turcica and mild horizontal tortuosity of the optic nerves. Magnetic Resonance Venography (MRV) further revealed bilateral narrowing of the sigmoid-transverse sinus junction. lumbar puncture was performed revealing a normal opening pressure of 16 cmH_2_O.

For both eyes, ffERG showed negative b-wave in the dark-adapted (DA) 10.0 cd s/m^2^ response and reduced cone response in light-adapted (LA) 30 Hz flicker, consistent with cone dysfunction, VF showed generalized loss, SD-OCT of the macula showed Ellipsoid Zone (EZ) loss and shaggy photoreceptors (Fig. 2.1), while SD-OCT of the optic nerve head showed increased Retinal Nerve Fiber Layer (RNFL) thickness (Fig. 3). FAF showed the beginning of a macular ring of hyper-autofluorescence. Color fundus photograph showed bilateral disc swelling and ring-like maculopathy.

**Fig. 2:**
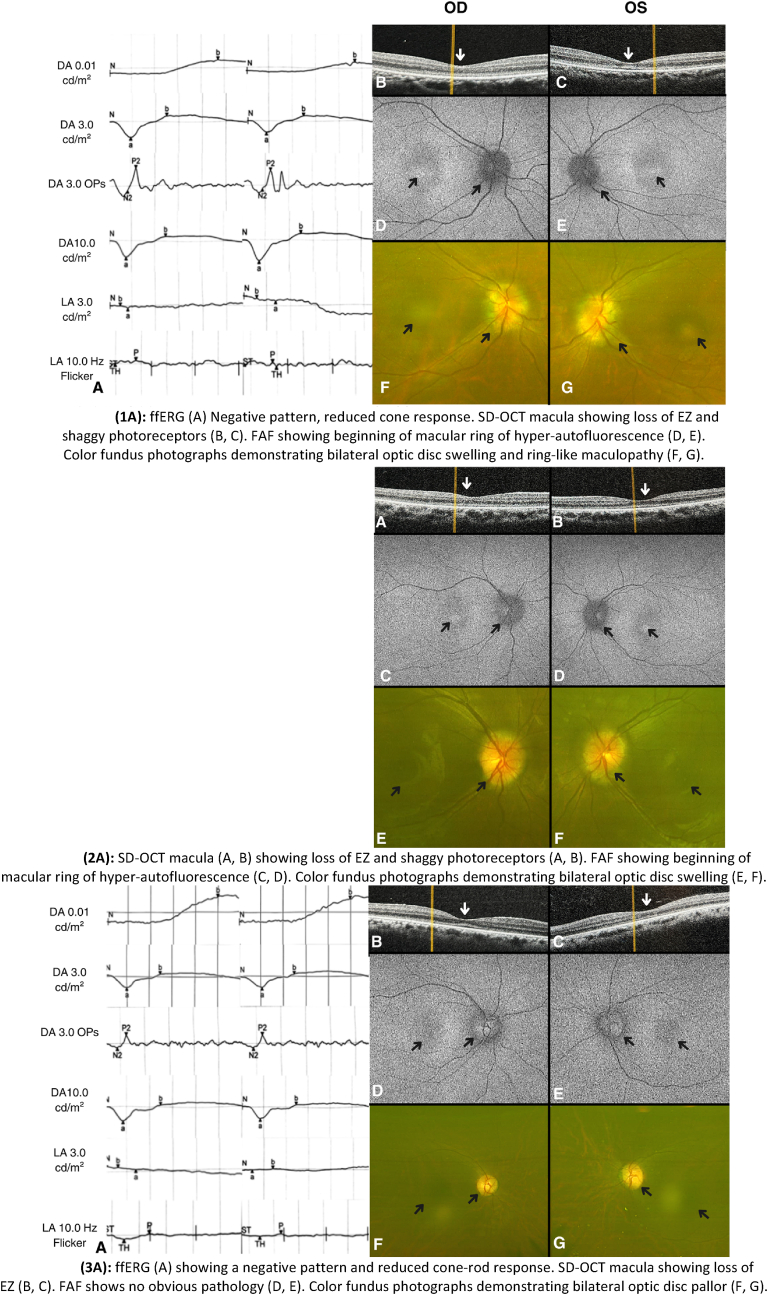
Imaging and ERG findings of family A (*DA: Dark Adapted, LA: Light adapted)* **(1A)**: ffERG (A) Negative pattern, reduced cone response. SD-OCT macula showing loss of EZ and shaggy photoreceptors (B, C). FAF showing beginning of macular ring of hyper-autofluorescence (D, E). Color fundus photographs demonstrating bilateral optic disc swelling and ring-like maculopathy (F, G). **(2A)**: SD-OCT macula (A, B) showing loss of EZ and shaggy photoreceptors (A, B). FAF showing beginning of macular ring of hyper-autofluorescence (C, D). Color fundus photographs demonstrating bilateral optic disc swelling (E, F). **(3A)**: ffERG (A) showing a negative pattern and reduced cone-rod response. SD-OCT macula showing loss of EZ (B, C). FAF shows no obvious pathology (D, E). Color fundus photographs demonstrating bilateral optic disc pallor (F, G). (For interpretation of the references to color in this figure legend, the reader is referred to the Web version of this article.)

**Fig. 3:**
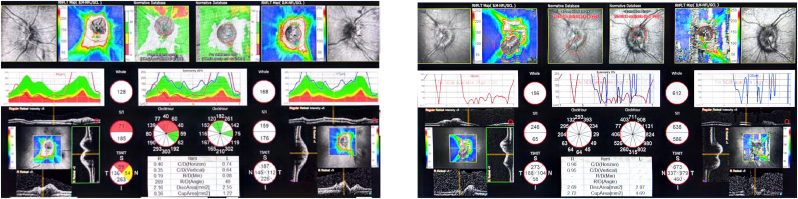
SD-OCT of the optic nerve showing bilateral thickening for case 1A (Left) and 2A (Right).

The patient was started on Acetazolamide at a dose of 500 mg twice daily for about 10 months before it was tapered off. However, after a five-month gap, symptoms recurred, necessitating reinitiation of the medication for another two months before it was stopped again.

#### 3.1.2 Case 2A

An 18-year-old female who is medically free with an old history of low vision since the age of 10-years-old presented with new onset of progressive headache, tinnitus, nausea, and photophobia. She was previously evaluated at the age of 10 years old, during which she was complaining of decreasing vision. At that time, BCVA was 20/100 for both eyes, and examination of her dilated fundus showed bilateral disc pallor. On her current visit with her sister (1A), BCVA was 20/100 in the right eye and 20/150 in the left eye. Automated refraction showed high myopia (7 diopters for the right eye and 6 diopters for the left eye) and astigmatism for both eyes. Intraocular pressure was 13 mmHg in the right eye and 17 mmHg in the left eye and slit-lamp examination of the anterior segment revealed no abnormalities. A dilated fundus examination showed bilateral optic disc swelling, classified as grade 3.^15^ BMI was 25.

MRI of the brain revealed horizontal tortuosity, prominent cerebrospinal fluid (CSF) cuffs around the optic nerves, and stenosis of the lateral segment of the right transverse sinus. A lumbar puncture was recommended but not performed due to the patient's refusal. VF testing demonstrated generalized visual field loss affecting the right eye more than the left. Similar to her sister, SD-OCT of the optic nerve head showed increased RNFL thickness (Fig. 3), while SD-OCT of the macula showed loss of the EZ and shaggy photoreceptors (Fig. 2.2). FAF showed the beginning of a macular ring of hyper-autofluorescence. Color fundus photograph showed bilateral optic disc swelling and ring-like maculopathy. The patient was not available for ERG testing. She was also treated with Acetazolamide 500 mg twice daily, resulting in the resolution of both headache and optic disc swelling. However, upon gradual discontinuation, both symptoms recurred, necessitating the resumption of the medication.

#### 3.1.3 Case 3A

A 33-year-old male who is medically free presented with a long-standing history of poor vision since childhood accompanied by photophobia, color blindness, and nyctalopia. BCVA was 20/400 for both eyes. Automated refraction showed myopia and astigmatism for both eyes. Total color blindness was noted on color-vision testing, with a score of 1/21. Upon dilated fundal examination, mottling of the Retinal Pigment Epithelium (RPE) was seen, and the optic disc appeared pale. SD-OCT of the macula showed loss of EZ outside the fovea in both eyes. SD-OCT of the optic nerve head showed thinning of the RNFL in the right (82) and left (86) eye. In ffERG, a reduced rod response in DA 0.01 cd s/m^2^ response and negative b-wave in the DA 10.0 cd s/m^2^ response, suggesting generalized retinal dysfunction. Additionally, reduced responses were observed in the LA 30 Hz flicker, consistent with cone system dysfunction. Color fundus photograph showed bilateral optic disc generalized pallor. (Fig. 2.3).

#### 3.1.4 Genetic analysis

Genetic analysis of the POC1B gene in the three affected siblings (1A, 2A, and 3A) identified a homozygous nonsense variant, NM_172240.3:c.672C > G (p.Tyr224∗), located in exon 6 of 12. This variant introduces a premature stop codon and is classified as pathogenic according to ACMG guidelines. In silico predictions indicated weak conservation of the site. Targeted sequencing confirmed the variant in all three cases, consistent with autosomal recessive inheritance. No other clinically relevant variants were detected.

### 3.2 Family (B)

#### 3.2.1 Case 1B (Proband)

**Fig. 4:**
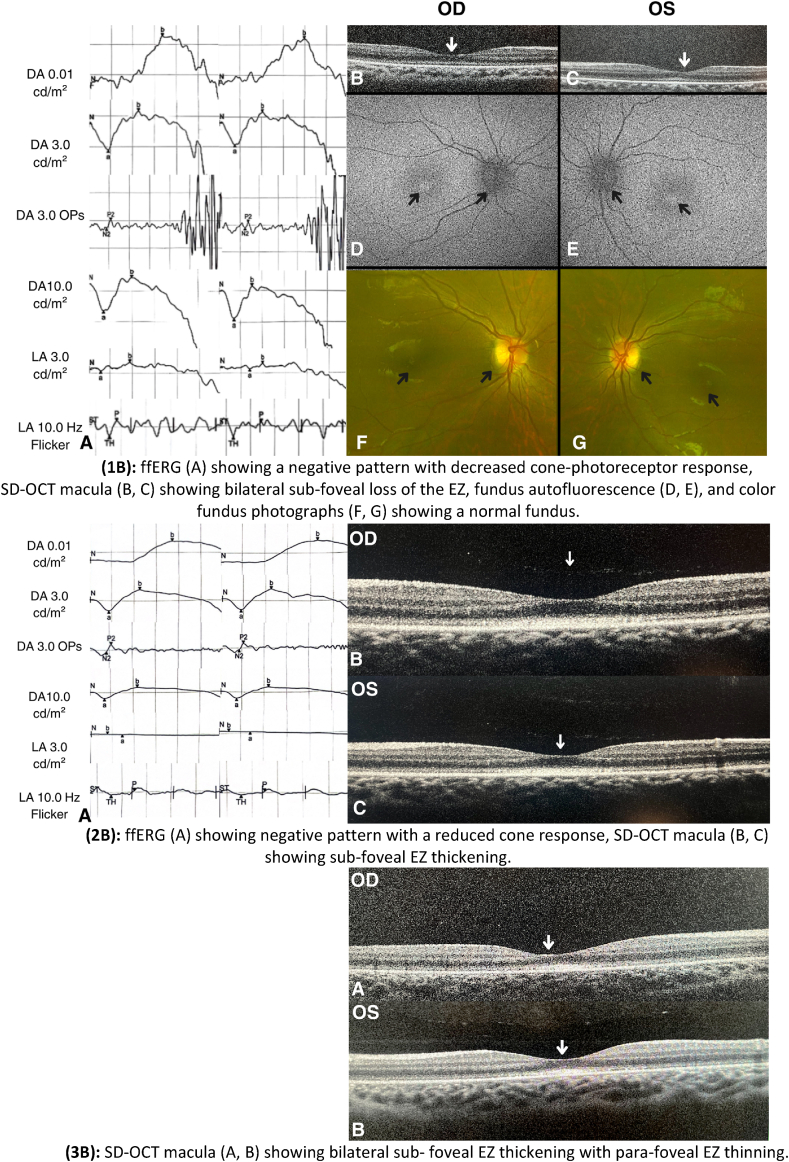
Imaging and ERG findings of family B (*DA: Dark Adapted, LA: Light adapted)* **(1B)**: ffERG (A) showing a negative pattern with decreased cone-photoreceptor response, SD-OCT macula (B, C) showing bilateral sub-foveal loss of the EZ, fundus autofluorescence (D, E), and color fundus photographs (F, G) showing a normal fundus. **(2B)**: ffERG (A) showing negative pattern with a reduced cone response, SD-OCT macula (B, C) showing sub-foveal EZ thickening. **(3B)**: SD-OCT macula (A, B) showing bilateral sub-foveal EZ thickening with para-foveal EZ thinning. (For interpretation of the references to color in this figure legend, the reader is referred to the Web version of this article.)

An 8-year-old female presented to our clinic reporting longstanding history of poor vision associated with photophobia. She is the child of a consanguineous marriage, with medical history including obesity and an ectopic right kidney. Her childhood development was otherwise normal. Family history was positive for visual impairment among second-degree maternal relatives from both genders, but none with confirmed evaluation and diagnosis. BCVA was 20/250 for the right eye and 20/400 for the left eye. Cycloplegic refraction revealed astigmatism only. No dysmorphic facies or extra digits were noted on examination. Fine horizontal nystagmus was noted in the left eye. Anterior segment examination was unremarkable, and dilated fundal examination showed normal optic discs with no macular or retinal pathologies. In ffERG, a negative b-wave was observed in the DA 10.0 cd s/m^2^ response and reduced cone response in LA 30 Hz flicker, consistent with cone dysfunction. SD-OCT of the macula revealed bilateral sub-foveal loss of the EZ, FFA showed beginning of macular ring of hyper-autofluorescence (Fig. 4).

#### 3.2.2 Case 2B

This is a 7-year-old female who was brought into the clinic by her parents for evaluation after her sister (case 1B) was diagnosed with cone-rod dystrophy. She also had history of decreased vision and photophobia. BCVA was 20/100 for both eyes. Cycloplegic refraction showed high myopia (4 diopters and 8 diopters for the right and left eyes, respectively) with mild astigmatism. She had full color vision 21/21 for both eyes. In line with her sister's (1B) ffERG, a negative b-wave was seen in the DA10.0 cd s/m^2^ response and reduced cone response in LA 30 Hz flicker, suggesting cone dysfunction. A sub-foveal EZ thickening was noted on SD-OCT of the macula as seen in Fig. 4.

#### 3.2.3 Case 3B

A 3-year-old boy who is known to have Glucose 6-phosphate-uridyl-transferase deficiency was brought into the clinic by his parents after the diagnosis of his older sister (1B) as they noted poor vision associated with photophobia since birth. BCVA was 20/30 and 20/50 for the right and left eye, respectively. Cycloplegic refraction revealed astigmatism only. Anterior segment examination was unremarkable. Dilated fundal examination showed a healthy optic disc and macula with no retinal abnormalities. ERG was not feasible due to difficulties in patient's cooperation during testing. SD-OCT of the macula revealed bilateral sub-foveal EZ thickening with para-foveal EZ thinning (Fig. 4.3).

#### 3.2.4 Genetic analysis

Genetic analysis of the POC1B gene in the three affected siblings (1B, 2B, and 3B) identified a homozygous frameshift deletion, NM_172240.3:c.991del (p.Arg331Glufs∗13), located in exon 9 of 12. This variant causes a frameshift leading to a premature stop codon and is classified as pathogenic according to ACMG guidelines. Targeted sequencing confirmed the presence of the variant in all three cases, consistent with autosomal recessive inheritance. No other clinically relevant variants were detected.

## 4 Discussion

This study presents a single generation from two unrelated families diagnosed with COD and CORD due to two distinct *POC1B* gene variants. A key finding is the unusual presence of bilateral optic disc swelling in two affected individuals.

In family A, a pathogenic nonsense variant in the *POC1B* gene was identified, while family B harbored a distinct pathogenic frameshift deletion affecting different codons of the same gene. This study adds further cases to a *POC1B* variant recently described as novel in Saudi Arabia.^16^ All variants are associated with autosomal recessive cone-rod dystrophy-20 (CORD20), characterized by primary cone degeneration followed by rod involvement. Among genes implicated in COD and CORD, *POC1B* remains as one of the least explored.^17^ A literature review conducted on January 20, 2025, using PubMed, Google Scholar, and Web of Science with the keywords “*POC1B*,” “cone dystrophy,” “cone-rod dystrophy,” “optic disc swelling,” and “papilledema” revealed no previous reports of *POC1B*-associated COD or CORD presenting with bilateral optic disc swelling.

Some phenotypic variability has been described in *POC1B*-related CORD. Durulu et al.^18^ followed 13 affected patients with a homozygous deletion mutation; all had reduced vision, photophobia, and dyschromatopsia. Despite electrophysiological rod involvement, nyctalopia was absent, with some reporting improved night vision. Our cohort showed early-onset disease with progression, consistent with prior reports.^19^^,^^20^ Visual acuity varied, with one study showing a range from −0.08 to 1.52 logMAR,^21^ suggesting a potentially variable and age-related progression rate.

The parafoveal hyperpigmented ring observed in Proband A resembles bull's-eye maculopathy (BEM), often seen in inherited retinal dystrophies such as those caused by *POC1B*, ABCA4, CRX, and GUCY2D mutations.^20^^,^^22^ BEM has also been associated with benign concentric annular macular dystrophy.^23^ In this context, BEM is considered a chronic finding related to central cone loss and longstanding RPE degeneration. While not pathognomonic, it suggests diagnoses such as macular dystrophy, COD, CORD, and RCOD.^24^ Fundus autofluorescence may not correlate with function, and patients may present with a normal fundal examination, highlighting the importance of electrophysiology and genetic testing.^24^^,^^25^ Differential diagnoses of BEM also include conditions like Stargardt disease, Bardet–Biedl syndrome, and drug-induced retinopathy from hydroxychloroquine or chloroquine.^22^^,^^24^

The co-occurrence of myopia and CORD, as seen in our patients, aligns with previous cases involving PROM1 mutations and X-linked high myopia.^26^^,^^27^ Proposed mechanisms include shared genetic pathways, altered visual feedback, and structural retinal changes.^24^^,^^25^

Proband B's OCT findings of blurred ellipsoid and interdigitation zones corresponds to those in prior *POC1B*-related CORD cases.^21^ Durulu et al.^18^ also noted inner and outer segment junction (IS/OS line) disruption in central and mid-peripheral retina with significantly reduced central foveal thickness. Findings like thinning of the photoreceptor layer, cone interdigitation zone (CIZ) absence, and EZ disruption are considered characteristic of *POC1B*-associated disease.^19^^,^^20^^,^^25^

In contrast to our cohort, which showed progressive, moderate to severe vision loss, a Japanese patient with the *POC1B*-related retinal dystrophy retained good acuity until his 60s, with mild changes on fundal examination, OCT, and ffERG.^28^ This suggests variable phenotypic expression and a possible age-related progression, as younger patients in Family B maintained better vision.

The optic disc swelling in our cases is atypical for COD/CORD and has only rarely been reported in other inherited retinal dystrophies like retinitis pigmentosa (RP).^11^, ^12^, ^13^, ^14^ Villa et al.^11^ described bilateral optic nerve head (ONH) edema in RP and Usher syndrome with unremarkable systemic workup. Cope et al.^12^ similarly reported chronic papilledema in a family with sector RP. Edema often progressed to optic atrophy and visual impairment with age.

Other reports link optic disc swelling to intracranial hypertension, as in a Bardet–Biedl case with elevated CSF pressure.^14^ In our patients, CSF opening pressure was borderline, suggesting probable rather than definite idiopathic intracranial hypertension (IIH). Nonetheless, imaging and response to acetazolamide supported the diagnosis. Zenteno et al.^20^ reported a similar case of *POC1B*-related CORD requiring shunting for IIH. In contrast, some cases showed no elevated pressure but still improved with acetazolamide,^13^ hinting at subclinical mechanisms.

Possible explanations include inflammation due to rapid degeneration of photoreceptors and RPE as in RP patients, who have shown presence of anterior vitreous cells and elevated inflammatory markers like MCP-1, known to activate microglia and promote photoreceptor apoptosis.^29^^,^^30^ Steroid responsiveness in RP-associated optic disc edema further supports this theory.^31^

Another hypothesis involves the role of *POC1B* in ciliopathies. *POC1B* localizes to photoreceptor cilia, and its dysfunction may affect CSF dynamics.^32^ Impaired ependymal cilia have been linked to hydrocephalus and papilledema.^33^^,^^34^ In murine models, defective CSF clearance via the cribriform plate resulted in increased intracranial pressure,^34^ offering a potential mechanistic link between *POC1B* mutations and optic disc swelling.

Finally, the presence of optic disc swelling in these cases may be linked to *POC1B* mutations, the underlying retinal dystrophy, or an unrelated etiology. Given that bilateral optic disc swelling is not a typical feature of COD/CORD, its association with *POC1B* remains uncertain. Further investigations are needed to determine whether this finding represents a novel aspect of *POC1B*-related COD/CORD or an independent pathology.

## 5 Conclusions

This study sheds light on a series of cases from two families with COD and CORD caused by two distinct variants of the *POC1B* gene. The unusual clinical presentation of optic disc swelling, which is not typically associated with retinal dystrophies, provides a valuable insight into the phenotypic variability of this condition -a point that physicians should be aware of. Further research is needed to investigate underlying mechanisms.

## CRediT authorship contribution statement

**Noha Bamardouf:** Writing – review & editing, Writing – original draft, Visualization, Resources, Investigation, Data curation. **Abdulrahman Alsaidi:** Writing – review & editing. **Faeeqah Almhmoudi:** Writing – review & editing, Validation, Supervision, Project administration, Methodology, Investigation, Conceptualization. **Nooran Badeeb:** Writing – review & editing, Validation, Supervision, Project administration, Methodology, Investigation, Conceptualization. **Enam Danish:** Writing – review & editing, Validation, Supervision, Project administration, Methodology, Investigation, Conceptualization.

## Patient consent

Written consent to perform genetic analysis has been obtained from patients. This report does not contain any personal identifying information. IRB ethical approval has been obtained.

## Authorship

All authors attest that they meet the current ICMJE criteria for Authorship.

## Funding

No funding or grant support.

## Declaration of competing interest

The authors declare that they have no known competing financial interests or personal relationships that could have appeared to influence the work reported in this paper.

## References

[1] American Academy of Ophthalmology (2016 Oct 3). https://www.aao.org/education/clinical-statement/guidelines-on-clinical-assessment-of-patients-with.

[2] Hamel C.P. (2007 Dec). Cone rod dystrophies. Orphanet J Rare Dis.

[3] Roosing S., Thiadens A.A., Hoyng C.B. (2014). Causes and consequences of inherited cone disorders. Prog Retin Eye Res.

[4] Gill J.S., Georgiou M., Kalitzeos A., Moore A.T., Michaelides M. (2019 May 1). Progressive cone and cone-rod dystrophies: clinical features, molecular genetics and prospects for therapy. Br J Ophthalmol.

[5] Roosing S., Lamers I.J.C., De V.E. (2014). Disruption of the basal body protein POC1B results in autosomal-recessive cone-rod dystrophy. Am J Hum Genet.

[6] Alfares A., Alkuraya F. (2016).

[7] El-Hazmi M.A., al-Swailem A.R., Warsy A.S., al-Swailem A.M., Sulaimani R., al-Meshari A.A. (1995). Consanguinity among the Saudi Arabian population. J Med Genet.

[8] Aboshiha J., Dubis A.M., Carroll J. (2016). The cone dysfunction syndromes. Br J Ophthalmol.

[9] Thiadens A.A., Phan T.M., Zekveld-Vroon R.C. (2012). Clinical course, genetic etiology, and visual outcome in cone and cone-rod dystrophy. Ophthalmology.

[10] Hamel C.P., Griffoin J.M., Bazalgette C. (2000). Molecular genetics of pigmentary retinopathies: identification of mutations in CHM, RDS, RHO, RPE65, USH2A and XLRS1 genes. J Fr Ophtalmol.

[11] Villa A.M., Anderson S.F., Abundo R.E. (1997 Mar 1). Bilateral disc edema in retinitis pigmentosa. Optom Vis Sci.

[12] Cope L.A., Van Teeters W., Borda R.P., Mccrary I.I.I.J.A. (1976 Jun 30). International Symposium on Fluorescein Angiography Ghent 28 March—1 April 1976.

[13] Gonzalez-Gonzalez L.A., Scanga H., Traboulsi E., Nischal K.K. (2021 Apr 1). Novel clinical presentation of a CRX rod-cone dystrophy. BMJ Case Reports CP.

[14] Saida K., Inaba Y., Hirano M. (2014 Sep 1). A case of Bardet-Biedl syndrome complicated with intracranial hypertension in a Japanese child. Brain and Development.

[15] Frisén L. (1982). Swelling of the optic nerve head: a staging scheme. J Neurol Neurosurg Psychiatry.

[16] Alzahem T.A., AlTheeb A., Ba-Abbad R. (2024 Jan 2). Phenotypic and genotypic features of POC1B-associated cone dystrophy. Ophthalmic Genet.

[17] Munir A., Khan I.U., Rashid A. (2024 Nov 22). A novel homozygous missense variant in POC1B causes cone dystrophy in a consanguineous Pakistani family. Ophthalmic Genet.

[18] Durlu Y.K., Köroğlu Ç., Tolun A. (2014 Oct 1). Novel recessive cone-rod dystrophy caused by POC1B mutation. JAMA Ophthalmol.

[19] Birtel J., Eisenberger T., Gliem M. (2018 Mar 19). Clinical and genetic characteristics of 251 consecutive patients with macular and cone/cone-rod dystrophy. Sci Rep.

[20] Zenteno J.C., Arce-Gonzalez R., Matsui R. (2023 Feb). Clinical-genetic findings in a group of subjects with macular dystrophies due to mutations in rare inherited retinopathy genes. Graefes Arch Clin Exp Ophthalmol.

[21] Kameya S., Fujinami K., Ueno S. (2019 Aug 1). Phenotypical characteristics of POC1B- associated retinopathy in Japanese cohort: cone dystrophy with normal funduscopic appearance. Investig Ophthalmol Vis Sci.

[22] Nasser F., Kurtenbach A., Kohl S., Obermaier C., Stingl K., Zrenner E. (2019 Aug 15). Retinal dystrophies with bull’s-eye maculopathy along with negative ERGs. Doc Ophthalmol.

[23] Miyake Y., Shiroyama N., Horiguchi M., Saito A., Yagasaki K. (1989 Jan 1). Bull's-eye maculopathy and negative electroretinogram. Retina.

[24] Kurz-Levin M.M., Halfyard A.S., Bunce C., Bird A.C., Holder G.E. (2002 May 1). Clinical variations in assessment of bull's-eye maculopathy. Arch Ophthalmol.

[25] Kominami A., Ueno S., Kominami T. (2018 Mar 4). Case of cone dystrophy with normal fundus appearance associated with biallelic POC1B variants. Ophthalmic Genet.

[26] Khan A.O., Bolz H.J. (2015 Dec 9). Pediatric cone-rod dystrophy with high myopia and nystagmus suggests recessive PROM1 mutations. Ophthalmic Genet.

[27] Young T.L., Deeb S.S., Ronan S.M. (2004 Jun 1). X-linked high myopia associated with cone dysfunction. Arch Ophthalmol.

[28] Hayashi T., Mizobuchi K., Kameya S., Ueno S., Matsuura T., Nakano T. (2023 Aug). A mild form of POC1B-associated retinal dystrophy with relatively preserved cone system function. Doc Ophthalmol.

[29] Yoshida N., Ikeda Y., Notomi S. (2013 Jan 1). Clinical evidence of sustained chronic inflammatory reaction in retinitis pigmentosa. Ophthalmology.

[30] Sachdev M.S., Verma L., Garg S.P., Moonis M., Shekar C.H., Gupta N.K. (1987 Jan 1). Bilateral disc oedema in retinitis pigmentosa--an unusual sign. Jpn J Ophthalmol.

[31] Patil-Chhablani P., Tyagi M., Kekunnaya R., Narayanan R. (2015 Aug 3). Acute unilateral vision loss with optic disc oedema in retinitis pigmentosa. Case Reports.

[32] Beck B.B., Phillips J.B., Bartram M.P. (2014 Oct). Mutation of POC 1 B in a severe syndromic retinal ciliopathy. Hum Mutat.

[33] Tay S.A., Vincent A.L. (2020 Jul 3). Senior-Løken syndrome and intracranial hypertension. Ophthalmic Genet.

[34] Xue Y., Gursky Z., Monte B. (2022 Mar 5). Sustained glymphatic transport and impaired drainage to the nasal cavity observed in multiciliated cell ciliopathies with hydrocephalus. Fluids Barriers CNS.

